# The Influence of Casein Coatings on the Corrosion Behavior of Mg-Based Alloys

**DOI:** 10.3390/ma15041399

**Published:** 2022-02-14

**Authors:** Aneta Kania, Katarzyna Cesarz-Andraczke, Zbigniew Brytan, Łukasz Reimann, Paulina Smolarczyk

**Affiliations:** 1 Department of Engineering Materials and Biomaterials, Faculty of Mechanical Engineering, Silesian University of Technology, Konarskiego 18a, 44-100 Gliwice, Poland; katarzyna.cesarz-andraczke@polsl.pl (K.C.-A.); zbigniew.brytan@polsl.pl (Z.B.); paulina.smolarczyk@polsl.pl (P.S.); 2 Materials Research Laboratory, Faculty of Mechanical Engineering, Silesian University of Technology, Konarskiego 18a, 44-100 Gliwice, Poland; lukasz.reimann@polsl.pl

**Keywords:** Mg-based alloys, casein coatings, conversion coatings, corrosion resistance

## Abstract

This article discusses the influence of conversion casein coatings with a thickness of about 20 µm on the structure and the corrosion behavior of two magnesium alloys: MgCa2Zn1 and MgCa2Zn1Gd3. Casein is a protein that, along with whey protein, is a part of milk. Casein coatings are appropriate for bone growth because they contain high amounts of calcium and phosphorus. In this work, casein coatings and casein-free coatings were applied on Mg-based alloys using the conversion process. The structure and topography observations were presented. The corrosion behavior was determined by electrochemical and immersion tests, and electrochemical impedance spectroscopy (EIS) in chloride-rich Ringer solution. The obtained results show that conversion casein coatings effectively protect Mg-based alloys against corrosion. This was confirmed by higher corrosion potentials (E_corr_), polarization resistances (R_p_) derived from Tafel’s and EIS analysis, as well as low hydrogen release. The volume of hydrogen released after 216 h of immersion for casein coatings applied to MgCa2Zn1 and MgCa2Zn1Gd3 alloys was 19.25 and 12.42 mL/cm^2^, respectively. The improvement in corrosion resistance of casein coatings was more significant for Mg alloy dopped with gadolinium. The lower corrosion rate of casein conversion coatings is explained by the synergistic effect of the addition of Gd in the Mg-based alloy and the formation of dense, tight conversion casein coatings on the surface of this alloy.

## 1. Introduction

In the field of medicine, magnesium alloys are currently being studied for their use in implantology, mainly in orthopedics. The biocompatibility of magnesium alloys, i.e., the chemical composition of metals present in the human body and the possibility of obtaining appropriate mechanical properties for implantology applications, is the main premise for continuous research in this respect. The biocompatibility of the chemical composition also likely enables the resorption, dissolution, and absorption of the degradation products of magnesium alloys in the human body. Properly designed Mg alloys are very promising materials for orthopedic implants. The development of this type of implant would not only eliminate the need to reoperate to remove the implant, but also reduce postoperative costs, including the costs of patient rehabilitation. Consequently, the development of Mg-based resorbable implants, among other biodegradable alloys [1,2], will significantly improve quality of life, increase patient comfort, and reduce overall treatment costs. However, the high degradation rate of magnesium alloys in the body fluid environment remains a problem. The high rate of implant degradation is associated with an intensive release of hydrogen. Constraints related to the release of too high a level of H_2_ from Mg-based alloys can be reduced by controlling the degradation rate of a resorbable implant [3,4]. The corrosion rate of magnesium alloys can be regulated in many ways. The corrosion resistance of magnesium alloys is also influenced by the microstructure–grain size and precipitates. Fine-grain alloys are generally more resistant to corrosion. In this work, two magnesium alloys, MgCa2Zn1 and MgCa2Zn1Gd3, were initially optimized in terms of corrosion resistance by selecting the appropriate alloying additives. The addition of rare earth metals, e.g., gadolinium, reduces the corrosion rate of magnesium alloys. Gd is characterized by high solubility in a solid solution of Mg at eutectic temperatures. The distribution of this element in the Mg matrix is responsible for decreases in the corrosion rate of Mg alloys. This is due to the formation of a new crystallographic β-phase, which is resistant to corrosion. The predominance of this phase in Mg alloys with Gd addition influence the increase in content of Mg(OH)_2_ that is formed on the surface of these alloys. This film slows down the dissolution of corrosion products, reducing the corrosion rate of magnesium alloys [5]. Hort et al. [6] investigated the corrosion behavior of Mg-Gd alloys (with 2, 5, 10 and 15 wt.% Gd) in 1 wt.% NaCl. They stated that the corrosion rate decreased significantly with the addition of Gd by up to 10%. As long as gadolinium is in a solid solution, the corrosion process slows down, and it was accelerated when precipitates were in the microstructure of Mg-Gd alloys. The addition of gadolinium to the MgCa2Zn1Gd3 alloy reduces the volume of eutectics, which is related to the lower proportion of the intermetallic phases Ca_2_Mg_6_Zn_3_ and Mg_2_Ca, which occur in the alloy [7]. The reduction of the Mg_2_Ca phase in the MgCa2Zn1Gd3 alloy, which accelerates the anodic kinetics of magnesium, improves resistance to corrosion. Gadolinium also reduces the microporosity of the Mg-based alloys. Zinc reduces the adverse effects of other elements, including Fe, Cu, and Ni, for corrosion resistance. Calcium has a positive effect on microstructure, causing grain refinement. Furthermore, calcium, zinc, and magnesium are biocompatible elements that naturally occur in the human body and can be metabolized and released naturally.

The solution for rapid degradation is to completely block the degradation process during the concrescence of the bones (approximately 6 months). Degradation can also be blocked by applying protective coatings to a magnesium alloy that meets the strength and working environment. In the literature on the subject, both resorbable (phosphates [8,9,10], resorbable polymers [11,12,13]) and unresorbable (oxides [14,15,16], nitrides [17], carbides [18]) protective coatings have been studied in this regard. In order to achieve a resorbable magnesium alloy for an orthopedic implant, resorbable protective coatings seem to be a more preferred choice. Calcium phosphates, such as hydroxyapatite, brushite, and monetite, were used for resorbable coatings [8,9,10]. The occurrence of defects, cracks, and technological defects determines the degradation rate of ceramic materials. Defective ceramics in contact with water can be completely destroyed. The inclusions of other phases are equally unfavorable for ceramic materials, leading to their degradation. These inclusions, in contact with water, accelerate aging and increase volume, which accelerates the degradation process. These processes also have a direct impact on the deterioration of the mechanical properties of ceramic materials. Resorbable polymers, such as PLA (polylactic acid), have also been considered as materials for protective coatings on magnesium alloys [13]. PLA is biocompatible, and its degradation products, such as lactic acid, are easily metabolized in the human body, showing no toxicity. In addition to synthetic polymers, there is also a group of so-called biopolymers. This group includes casein [19]. In this work, casein was proposed as one of the bath ingredients for the preparation of protective coatings. Casein is one of the groups of milk proteins. Proteins belong to the group of biopolymers where the monomers are amino acids [20]. Mechanisms of casein degradation have not yet been investigated, but it is worth underlying that the casein is a group of phosphoproteins that has the ability to bind water molecules. This effect is called hydration. Initially, they swell and then dissolve to form colloidal particles [21]. The approximate elemental composition of casein is the following: C (53%), H (7%), O (22%), N (15.65%), S (0.76%), and P (0.85%) [22,23]. The authors of this study selected casein as a component of the coatings due to its probable non-toxicity to the human body (considering the chemical composition and current use of casein). Moreover, the authors assume that the degradation mechanisms of the casein coating will probably be similar to the degradation mechanisms of resorbable polymers already used in medicine (e.g., PLA as a material for surgical sutures) and thus beneficial because of the use of the material in medicine.

In the literature on the subject, there are few reports on the use of casein as a material for coatings for medical implants. Qin et al. [23] applied layers of casein and chitosan onto the Co-Cr-Mo alloy. The authors indicate that the tested casein-chitosan coatings did not show cytotoxicity. However, they indicate the bioactive properties of the studied coatings, consisting of an increased amount of calcium phosphates on the surface of the samples. In turn, Kumar et al., in their research [24], used a casein as a material for bone tissue engineering (material for bone grafts). The use of casein reduced porosity and therefore increased the mechanical properties of the bone graft. Casein, in studies [19], was also used as a nanogel ingredient for the treatment of skin cancer. 

In this work, coatings with casein (CN) and casein-free coatings (NZ) were applied on the surfaces of MgCa2Zn1 and MgCa2Zn1Gd3 alloys by a conversion method (dip coating). Baths consisting of NaOH, Zn(NO_3_)_2_, and casein were used to prepare protective coatings. Both NaOH and Zn(NO_3_)_2_ are necessary to obtain dense and uniform coatings on Mg alloys. In [9], NaOH was used to improve the corrosion resistance of Mg alloys. In turn, Zn(NO_3_)_2_ was added to the phosphatizing baths to deposition Zn or ZnO on the surface of the Mg alloys [25].

The aim of the study is to apply a dense and tight conversion coating with the addition of casein and casein-free coatings on two magnesium base alloys, MgCa2Zn1 and MgCa2Zn1Gd3, and assess their corrosion performance in a chloride-rich solution that simulates a physiological environment. Furthermore, the objective of the study is to verify whether casein-doped coatings are characterized by higher corrosion resistance compared to casein-free coatings.

## 2. Materials and Methods

**Figure 1:**
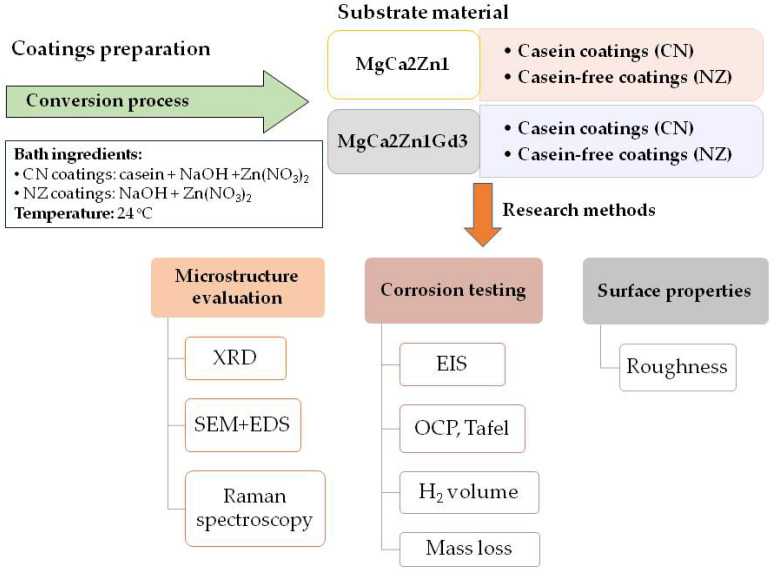
Diagram of research performed in the work.

The research methodology presented in the work is depicted in Figure 1.

The two base magnesium-calcium-zinc alloys, MgCa2Zn1 and MgCa2Zn1Gd3, were used as substrate materials for the deposition of conversion coatings with casein. The Mg-based alloys were obtained by induction melting at 750 °C with an Ar atmosphere. For the casting process, biocompatible elements with high purity (99.9%) were used. The molten alloys from chamotte-graphite crucibles were cast into sand molds. Samples of the alloys studied in the form of cylinders with diameters of 13 mm and heights of 6 mm were the substrate materials for deposition of the coatings. Before the investigations were carried out, the surfaces of all samples were mechanically polished with SiC paper, grade 800 to 4000, and then polished with a diamond suspension. Finally, they were ultrasonically degreased in acetone, cleaned in alcohol, and washed with distilled water. 

For the conversion coatings preparation, two baths were used. The casein coating (CN) was prepared using three compounds: casein (30 g·dm^−3^), NaOH (0.15M), and Zn(NO_3_)_2_ (0.07M). First, the casein was dissolved with NaOH solution. Zn(NO_3_)_2_ was added to the prepared mixture and mixed for 1 h. The second coating without casein (NC) was prepared from a mixture of two compounds: NaOH (0.15M) and Zn(NO_3_)_2_ (0.07M).

Both prepared conversion coatings with and without casein were applied to MgCa2Zn1 and MgCa2Zn1Gd3 alloys using a conversion process. The process was performed at room temperature with a deposition time of 12 h. 

The thickness of the conversion coatings was measured by an altitude profile using a confocal microscope. The average thickness of casein and casein-free coatings is 20 and 22 µm, respectively.

### 2.1. Structure Study and Phase Analysis

Surface morphology observations were made with a Zeiss (SUPRA, 35 model) scanning electron microscope (SEM, Thornwood, New York, NY, USA) (EHT = 3.0; 5.0 and 15.0 kV; SE mode) equipped with an energy-dispersive X-ray spectrometer (EDS) detector. The EDS analysis of the samples’ surface was performed in accordance with BS ISO 22309:2011 standard.

The samples’ topography and roughness measurements were performed using the ZEISS LSM Exciter 5 (Zeiss Company, Oberkochen, Germany) confocal microscope with an observation system of 4 lasers of wavelengths ranging from 405 to 633 nm. It was equipped with a ZEN image acquisition and analysis system. Roughness measurements were made in accordance with BS EN ISO 25178-607:2019 standard.

The phase analysis of the coatings was carried out with a PANalytical X’Pert PRO X-ray diffractometer (PANalytical, Almelo, The Netherlands), using Co Kα radiation. Analysis was carried out with the step registration method in a 2θ angular range of 30° to 100°. X-ray qualitative analysis was performed with HighScore Plus software (3.0e version) using a dedicated PAN-ICSD (Inorganic Crystal Structure Database) phase identification card database. XRD analysis was performed in accordance with EN 13925-1:2003, EN 13925-2:2003, and EN 13925-3:2005 standards.

Structure identification of the casein coatings was also performed with the Raman spectrometer (Renishaw, New Mills, UK, inVia Reflex model), which was equipped with an Ar ion laser with the wavelength of 514.5 nm. It was calibrated before each set of measurements using the line of Si at 520 cm^−1^ as a reference.

### 2.2. Electrochemical and Immersion Tests

Electrochemical corrosion tests were performed using an Autolab PGSTAT302N Multi BA potentiostat (Metrohm AG, Herisau, Switzerland). A saturated calomel electrode was the reference electrode, and a platinum rod was the counter electrode. The experiment was carried out in Ringer chloride-rich solution (8.6 g·dm^−3^ NaCl, 0.3 g·dm^−3^ KCl, 0.48 g·dm^−3^ CaCl_2_·6H_2_O) at a temperature of 37 °C. Polarization tests were carried out according to the following parameters: sample polarization from −0.25 V (from the average potential value of the open circuit potential test preceding the measurement) to +0.25 V (from the average potential value of the test), step of potential change (1 mV), speed of potential change (1 mV·s^−1^). Before measurements were taken, the samples were immersed in Ringer solution for 10 min for stabilization. The corrosion parameters (corrosion potential: E_corr_; corrosion current density: i_corr_; corrosion polarization resistance: R_p_) were determined using Tafel’s analysis. Electrochemical corrosion tests were performed in accordance with ISO 17475:2005 standard.

In addition, immersion tests were performed to estimate the gas corrosion product (volume of evolution of H_2_) for casein and casein-free coatings and uncoated alloys. Studies were carried out in Ringer solution at 37 °C for 48 h (for casein-free coatings and uncoated alloys) and 216 h (for casein coatings). The volume of hydrogen released was measured at the experimental test stand. The samples of Mg-based alloys and the alloys with applied coatings were put into glass beakers filled with Ringer solution. Each beaker was placed in a water bath heated to 37 °C. A funnel was placed over the sample to ensure that total hydrogen was collected. A burette filled with Ringer solution was placed above the funnel. The hydrogen released went into the burette and displaced the solution. The volume of released hydrogen is measured by reading the level of the Ringer solution in burette. The volume of the H_2_ evolution was measured and calculated, considering the frontal area of the samples. Mass loss measurements (P_w_, mm·y^−1^) were performed after 48 h of immersion in Ringer solution. The corrosion products were removed from the surface of the samples by immersion in CrO_3_ solution at 24 °C. The samples were then washed with distilled water and dried with air. The samples were weighed on an analytical balance by Radwag company (AS 310/X model, Radom, Poland) with an accuracy of 0.1 mg.

Electrochemical impedance spectroscopy (EIS) measurements were performed at room temperature (24 °C) to determine the electrical characteristics of the conversion coating. This was carried out by recording changes in resistance and impedance in the variable frequency range from 100 kHz to 0.01 Hz by the signal of 10 mV in Ringer solution. Based on the results, the Bode and Nyquist relationship was determined.

Cylindrical samples, with a testing area of 1.3 cm^2^, were prepared for electrochemical studies and immersion tests. After immersion studies, corrosion products of casein coatings and coatings without casein were investigated via SEM.

## 3. Results and Discussion

The use of Mg-based alloys in implantology is limited due to their poor corrosion resistance in the environment of body fluids. This leads to a deterioration of the mechanical properties of Mg alloys before the new tissues are properly and adequately treated. The corrosion resistance of magnesium alloys can be significantly improved with conversion protective coatings, hence the idea of applying two different coatings with casein (CN) and without casein (NZ) to the MgCa2Zn1 and MgCa2Zn1Gd3 alloys. 

Casein is a source of calcium and phosphorus. Calcium determines the proper condition of bones and teeth, and phosphorus is necessary for its proper absorption. Phosphorus and calcium are involved in the regulation of the bone mineralization process. Therefore, the use of casein in protective coatings for magnesium alloys as an orthopedic implant material is justified. In addition, casein is an environmentally friendly inhibitor of corrosion and is also an inexpensive, available natural polymer. It has one more important feature: the self-healing property. Yabuki et al. [26] evaluated the self-healing properties of casein coatings from the corrosion behavior of a sample in which a defect had been created. The coating consisting of TiO_2_ particles and casein caused an increase in polarization resistance and a decrease in corrosion current [26].

**Figure 2:**
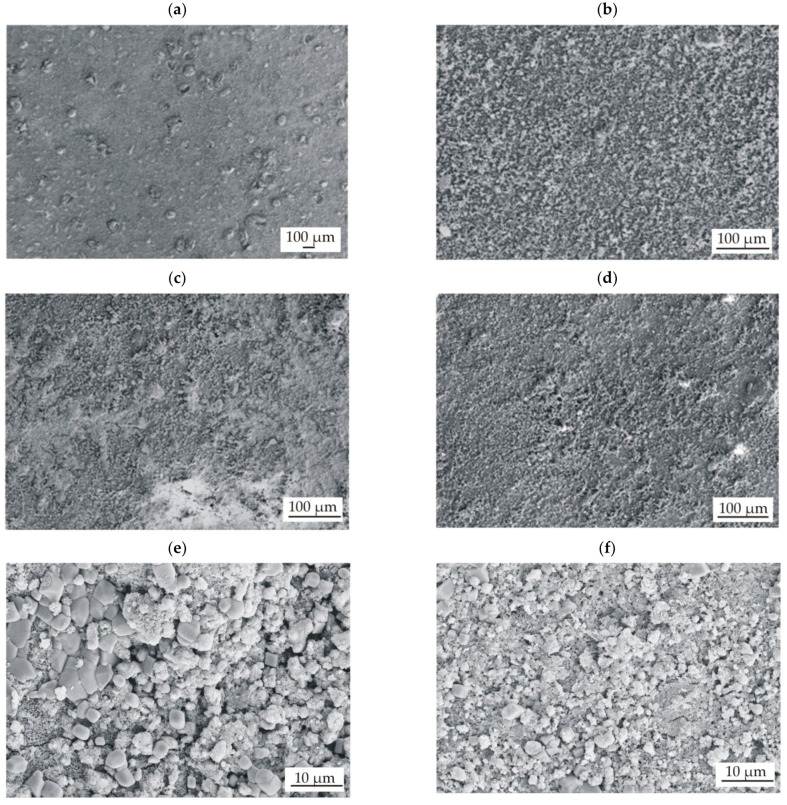
SEM images of surface morphology of conversion coatings deposited on MgCa2Zn1 alloy: (**a**) casein-free coating (NZ); (**c**,**e**) casein coating (CN) and MgCa2Zn1Gd3 alloy, respectively: (**b**) casein-free coating (NZ); (**d**,**f**) casein coating (CN).

**Figure 3:**
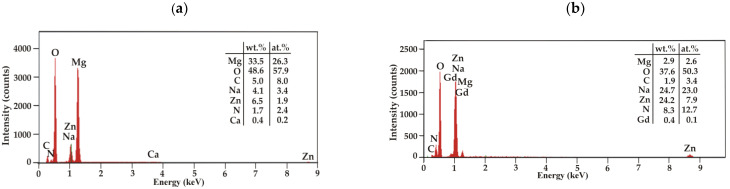
EDS analysis for casein coatings applied to (**a**) MgCa2Zn1 and (**b**) MgCa2Zn1Gd3 alloys.

The surface topography of conversion coatings with casein (CN) and without it (NZ), deposited on two Mg-based alloys, shows similar heterogeneous surface morphology (Figure 2). Single particles and their agglomerates can be seen on the surfaces of all prepared conversion coatings. It can also be observed that the casein coatings are more compact and less porous (Figure 2c,d). The EDS analysis of the casein films applied to the MgCa2Zn1 and MgCa2Zn1Gd3 alloys was performed (Figure 3). The analysis indicated the presence of Mg, O, C, N, Na, and Zn elements in both CN coatings and Ca for casein coating deposited on MgCa2Zn1 alloy. The presence of gadolinium in the casein coating applied to the MgCa2Zn1Gd3 alloy probably results from a relationship between gadolinium and casein, according to the high affinity of Gd^3+^ for phosphate, citrate, and carbonate ions. Gadolinium ions may compete with metal ions present in the human body, including Mg^2+^, Ca^2+^, Na^+^, K^+^, Fe^3+^, and Zn^2+^, creating strong ionic bonds [27,28]. It is worth mentioning that gadolinium cations can compete with Ca^2+^ in all biological systems that require Ca for proper function. Lanthanide ions can bind with much a higher affinity to calcium-requiring proteins. When bound to a Ca^2+^ binding enzyme, the replacement of Gd^3+^ often modifies the structure [29]. For this reason, it can be assumed that the Gd oxides present on the Mg alloy surface support the formation of a casein-enriched conversion coating with a compact, tight, and dense structure.

**Figure 4:**
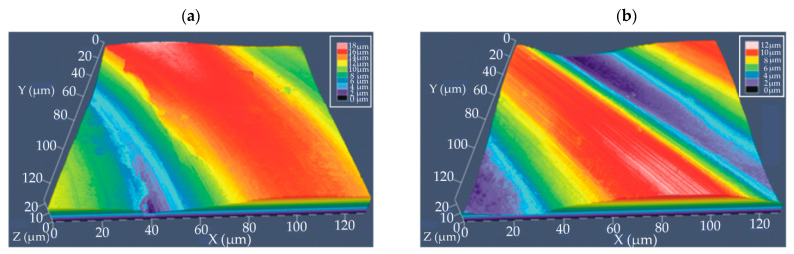

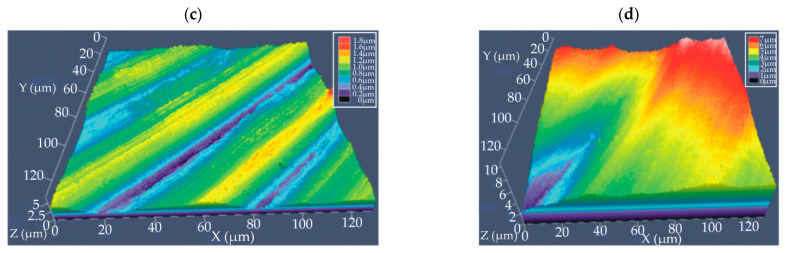
Confocal images of conversion coatings surface topography deposited on MgCa2Zn1 alloy: (**a**) casein coating (CN); (**b**) casein-free coating (NZ), and MgCa2Zn1Gd3 alloy: (**c**) casein coating (CN); (**d**) casein-free coating (NZ).

The results of the SEM observations were complemented by surface topography using a confocal microscope (Figure 4). Analysis of the coating topography was performed in an area of 120 µm^2^. The coatings were similar in their topography for both Mg-based alloys. It was observed that the surfaces of the coatings with and without casein were wavy (Figure 4). Furthermore, casein coatings applied on both MgCa2Zn1 and MgCa2Zn1Gd3 alloys were characterized by lower unevenness compared to conversion coatings without the casein component. This agrees with microscopic observations of topography, where the casein coatings (CN) were more compact and smooth. During confocal imaging, surface roughness analysis was performed, and surface roughness parameters were determined, including roughness average R_a_ and root mean square roughness R_S_ (Table 1). The roughness measurements show that the roughness values for conversion coatings applied to the MgCa2Zn1Gd3 alloy were lower compared to the coatings applied to the MgCa2Zn1 alloy. Furthermore, the lowest value of roughness measurements for the casein coating applied to the MgCa2Zn1Gd3 alloy probably is due to gadolinium content, which caused the reduction of microporosity resulting from the shrinkage of this alloy during casting. The advantage of conversion coatings is the covering of surface irregularities. In addition, higher affinity of the lanthanide ion to calcium, the creation strong ionic bonds, and the modification of a structure probably cause the formation of a smooth and compact casein coating.

**Figure 5:**
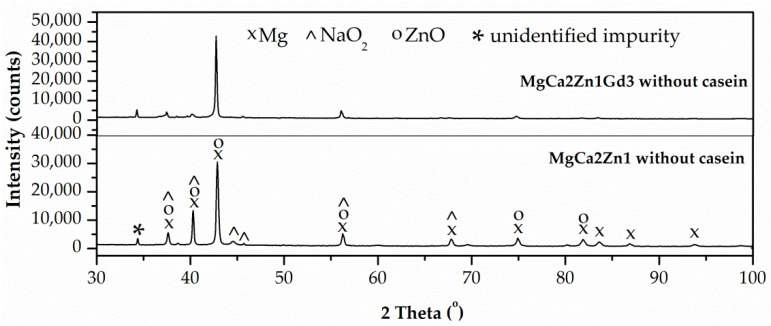
X-ray diffraction patterns of the casein-free conversion coating applied onto MgCa2Zn1 and MgCa2Zn1Gd3 alloys.

**Figure 6:**
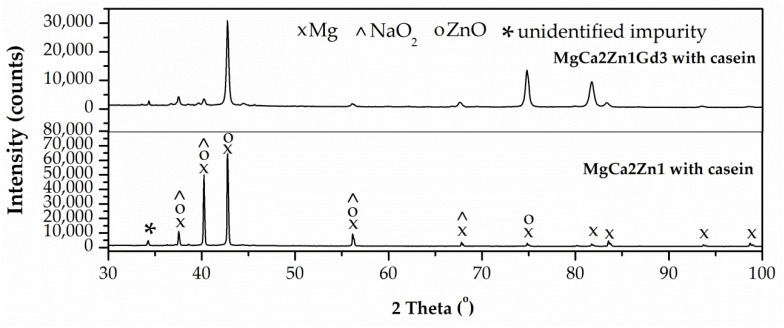
X-ray diffraction patterns of the conversion casein coatings applied onto MgCa2Zn1 and MgCa2Zn1Gd3 alloys.

The results of the X-ray phase analysis indicated the crystalline structure of the deposited coatings with casein and the casein-free ones (Figure 5 and Figure 6). In the diffraction patterns, in addition to the strong diffraction peaks from magnesium (at 37.55, 40.17, 42.79, 56.15, 74.79, 81.80, and 83.56 degrees of the 2θ angle)–JCPDS card No. 98-007-6259, there also were characteristic peaks from NaO_2_ (at 37.91, 39.62, 44.15, 45.33, 55.99 and 67.57 degrees of the 2θ angle)–JCPDS card No. 98-008-7181, ZnO (37.03, 40.19, 42.33, 55.79, 74.51 and 82.27 degrees of the 2θ angle)–JCPDS card No. 98-005-7478, and unidentified impurity from bath ingredients used in conversion process (at 34.22 degrees of the 2θ angle). On the basis of the phase analysis, it can be stated that on the Mg-based alloy surfaces, coatings composed of NaOH and Zn(NO_3_)_2_ were deposited. 

**Figure 7:**
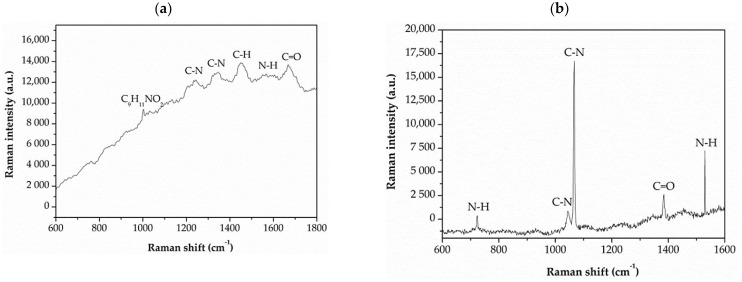
Raman spectra of the casein (**a**) and the casein coating deposited on MgCa2Zn1 (**b**).

The presence of casein in the CN coatings was confirmed by Raman spectroscopy (Figure 7). Raman analysis was also performed for the casein used in conversion coating preparation (Figure 7a). The recorded Raman spectra are characterized by a fluorescent background that partially covers the Raman bands. This is a common phenomenon in the case of polymeric materials and results from the induction of electronic transitions during sample excitation by radiation in the visible range. However, bands at Raman shifts at 1230, 1302, and 1522 cm^−1^ were identified. They were related to coupled stretching vibrations of C–N bonds and bending vibrations of N–H bonds of the peptide groups. The band recorded at 1444 cm^−1^ is related to the bending vibrations. The band occurring at the 1660 cm^−1^ is mainly related to the stretching vibrations of the C=O bond, while Raman peaks at 1005 cm^−1^ are derived from phenylalanine (C_9_H_11_NO_2_), the basic building block of most naturally occurring proteins, including casein. 

The Raman spectra for the casein coatings applied on both magnesium alloys were very similar. Therefore, in Figure 7b, an exemplary casein coating applied on the MgCa2Zn1 alloy was shown. The Raman spectrum of casein coatings shows the peak characteristic for C–N bonds (at 1055 and 1074 cm^−1^), and N–H bonds (at 720 and 1522 cm^−1^) of the peptide groups. The bands associated with C=O (at 1386 cm^−1^) were also present. 

In this work, corrosion investigations consisting of electrochemical and immersion tests were performed. The corrosion rate of magnesium alloys with the addition of various alloying elements in solutions containing chloride ions is well described in the literature [30]. The determination of the corrosion rate of Mg-based alloys is typically made by Tafel extrapolation of polarization curves, is derived from electrochemical impedance spectroscopy (EIS), or can be measured from the evolved hydrogen. Moreover, corrosion rates measured by the weight loss method are frequently used, and obtained results are typically in good agreement with the corrosion rate measured using hydrogen evolution in a chloride solution. Thus, both techniques provide a reliable measurement of the steady-state corrosion rate of the Mg-based alloy. However, in the case of electrochemical measurements, such good compliance is not provided for the steady-state corrosion rate of Mg alloys. In general, electrochemical measurements give corrosion rates for Mg-based alloys less than the steady-state corrosion rates measured by weight loss in immersion tests. For this reason, three methods of determining corrosion behavior were used (immersion test, Tafel extrapolation, and EIS).

**Figure 8:**
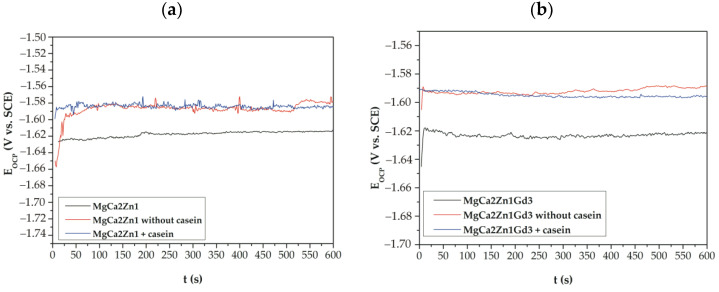
The E_OCP_ changes as a function of immersion time for studied casein and casein-free coatings applied to: (**a**) MgCa2Zn1 and (**b**) MgCa2Zn1Gd3 alloys in Ringer solution at 37 °C.

Measurement of changes in open circuit potential (E_OCP_) was used to evaluate protective properties of casein and casein-free coatings applied to the MgCa2Zn1 and MgCa2Zn1Gd3 alloys. The curves determined for a stationary potential as a function of immersion time indicate that both casein and casein-free coatings applied to the studied alloys show a slight activity in a chloride-rich environment (Figure 8), especially in cases of conversion coatings applied to the alloy without gadolinium addition. Furthermore, the CN coating applied to the MgCa2Zn1Gd3 alloy is more stable in the Ringer solution than the same coating applied to the second alloy. It can also be observed that the studied alloys were characterized by slight fluctuations.

**Figure 9:**
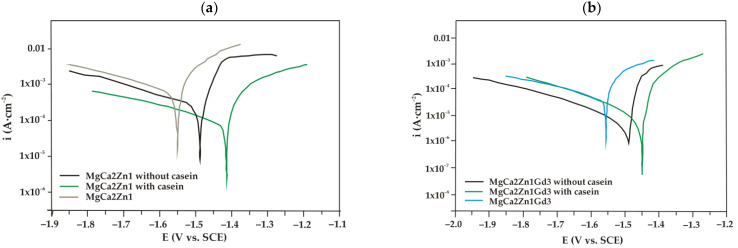
Polarization curves for the casein coating (CN), casein-free coating (NZ), and uncoated Mg-based alloys in Ringer solution at 37 °C, (**a**) MgCa2Zn1 alloy; (**b**) MgCa2Zn1Gd3 alloy.

The values of E_OCP_ potential are very similar for all of the coatings studied. The very small differences in the E_OCP_ potential between casein and casein-free coatings result from changes in their chemical composition, but these changes are not greater than 10 mV. Furthermore, it can be observed that cathodic polarization shifts the corrosion potential (E_corr_) toward more positive values (Figure 8 and Figure 9). The increase in corrosion potential (about 130 and 140 mV for casein coatings applied to both alloys; for example, E_OCP_ for the CN coating on MgCa2Zn1Gd3 alloy is about 1580 mV and E_corr_ is 1450 mV, and about 90 and 100 mV for casein-free coatings) suggests that casein and casein-free coatings exhibit good corrosion resistance.

The potentiodynamic curves in electrochemical studies give the basis information on the corrosion mechanism of Mg alloys with a conversion coating enriched with casein. Polarization curves of casein coatings (CN), casein-free coatings (NZ), and uncoated MgCa2Zn1 alloy (Figure 9a) were located in a higher current range (between 10^−2^ and 10^−3^ A·cm^−2^), than the same for MgCa2Zn1Gd3 alloy (Figure 9b), which were located in the current range 10^−3^–10^−4^ A·cm^−2^. The location of the curves analyzed indicated a higher corrosion resistance of the substrate MgCa2Zn1Gd3 alloy and both conversion coatings (CN, NZ) applied to it. 

It can also be observed that coatings applied on both Mg alloys were characterized by a higher corrosion resistance compared to the uncoated alloys. This was confirmed by Tafel extrapolation of polarization curves (Table 2), where the higher corrosion potentials (E_corr_) and polarization resistances (R_p_), and the lower values of corrosion current densities (i_corr_) were determined for samples with conversion coatings, compared to uncoated Mg alloys. 

Guo et al. [31] also studied an improvement in the biocorrosion resistance of the Mg alloy with deposited coatings [31]. They fabricated, using chemical conversion and deep-coating methods, a composite calcium phosphate (CaP)/collagen (Col) coating. The Col coating was stated to seal the pores and cracks of the CaP coating and the composition of CaP/Col significantly reduced the degradation rate of the studied alloy. Cordoba et al. [32] showed that the collagen and chitosan layer on the top of the silane-TiO_2_ coating provided additional corrosion protection. Similar studies carried out for magnesium alloys have confirmed the effectiveness and function of biopolymers (i.e., chitosan, cellulose, collagen) in terms of corrosion resistance, biodegradability, and biocompatibility [13,33,34,35]. It seems reasonable to say that natural polymers, such as casein, are promising biomaterials for the surface modification of Mg alloys.

**Figure 10:**
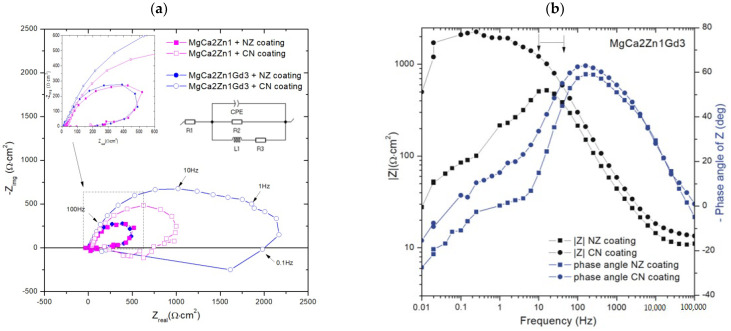
EIS results in (**a**) Nyquist plot of experimental data of conversion coatings on Mg-based alloys and adopted EEC circuit; (**b**) Bode plot of fitted data of casein (CN) and casein-free (NZ) coatings on MgCa2Zn1Gd3 alloy.

The interpretation of EIS measurements involves the use of an electrical equivalent circuit (EEC) as shown in the Nyquist plot in Figure 10a. The use of this type of EEC for magnesium-based alloys can be found in the literature [36,37]. Element R1 corresponds to the electrolyte resistance, that is, the Ringer solution. R2 may be attributed to the resistance to charge transfer at the phase interface. The constant phase element (CPE) can be related not only to the film or passive film thickness but also to the local dielectric constant. The CPE parameters and n may be interpreted in terms of these physical properties. CPE has numerous interpretations and can be associated with the surface roughness, porosity, inhomogeneous reaction rates on a surface, nonuniform current distribution, and specific character of the coating composition. The values of CPE element cannot be linked to the capacitance of the whole coating present on the surface of the magnesium alloys. They can be associated with a thin MgO film at the metal coating interface. The CPE parameter determined in the EIS tests serve only for the purpose of a better adjustment of the system and is informative in this case, while the main anticorrosion properties are assessed on the basis of the polarization resistance of the EIS test. An inductor, L1, and a resistance, R3, (inductance resistance) were also included to represent the inductive response that appears at low frequency [38]. The phase angle versus frequency in the Bode representation (Figure 10b) shows a peak at the frequency range of 100–1000 Hz for both casein-free and casein coatings, and then gradual decreases to 0° and below to −20° of phase angle at low frequency, below 10 Hz. The casein doped coating exhibits a larger impedance modulus and phase angle over a wide range of frequencies compared to the casein-free samples. The impedance at frequencies below 100 Hz gradually increases when casein is present in the coating, proving a higher corrosion resistance, while for magnesium alloy coated with casein-free conversion coating, there is peak at 10–100 Hz and then decreases at lower frequencies.

The corrosion resistance of conversion coatings was compared using the values of the polarization resistance R_p(EIS)_, which for used equivalent circuit was calculated as a sum of resistances, 1/R_p_ = 1/R2 + 1/R3. It was found that conversion coating with casein shows higher R_p(EIS)_ than casein-free coatings (Table 3). Moreover, it was noted that Mg alloy dopped with Gd shows higher polarization resistance. Obtained results are in good agreement with the polarization studies. 

These results of the electrochemical studies agreed with the results of the roughness measurements, where the R_a_ and R_S_ values for the casein coatings were lower compared to the roughness values obtained for the casein-free coatings. A similar relationship can be found in studies on Mg and Mg alloys [39,40], where corrosion resistance decreases with increasing surface roughness. Walter et al. [40] investigated the influence of surface roughness on corrosion resistance and pitting corrosion behavior of the AZ91 alloy in a chloride-rich environment. The authors stated that the corrosion current and the pitting tendency of the Mg-based alloy were increased with increasing surface roughness. The works [39,40] also indicated that surface roughness plays an essential role in the passivation behavior of magnesium and its alloys.

Electrochemical studies also show that the polarization resistances of the casein coatings (CN) were also higher compared to those of the casein-free conversion coating (NZ). In the case of MgCa2Zn1 alloy, polarization resistance was doubled in respect to uncoated substrate, while for gadolinium-alloyed MgCa2Zn1Gd3, the transition resistance (R_p_) between the surface and the electrolyte increased even more effectively (ca. 6 times), from 363.1 Ω·cm^2^ of uncoated substrate to 2137 Ω·cm^2^ for casein-enriched conversion coating. Similar confirmation was obtained in the EIS tests where R_P(EIS)_ for coatings with casein was higher than for conversion coatings without its addition. The synergistic effect of the addition of gadolinium in magnesium MgCa2Zn1Gd3 alloy and higher corrosion resistance of deposited casein coating can be observed. The higher corrosion resistance of the casein coating in this case may be related to trivalent lanthanide cations, where gadolinium cations Gd^3+^ can compete with metal ions present in the body [27,28]. For this reason, it can be assumed that the Gd oxides present on the Mg alloy surface support the formation of a casein-enriched conversion coating with a compact, tight, dense structure. 

**Figure 11:**
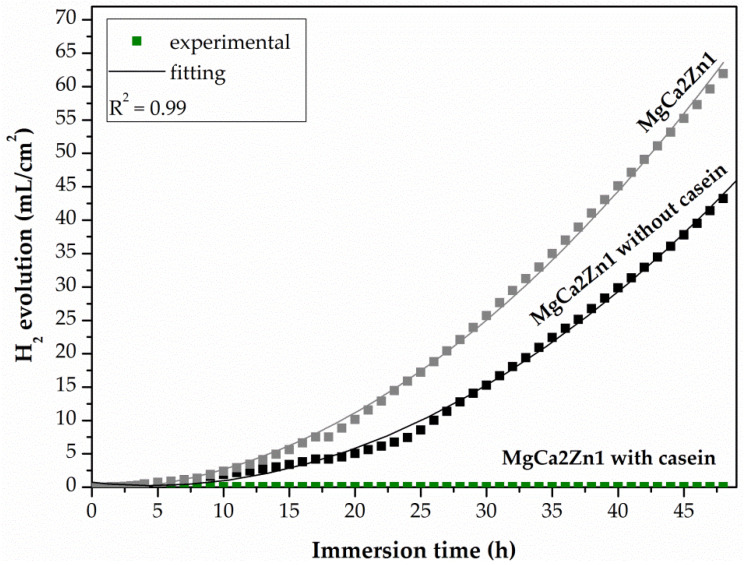
Volume of hydrogen evolution as a function of immersion time in Ringer solution at 37 °C for 48 h for the coatings applied to the MgCa2Zn1 alloy and uncoated alloy.

**Figure 12:**
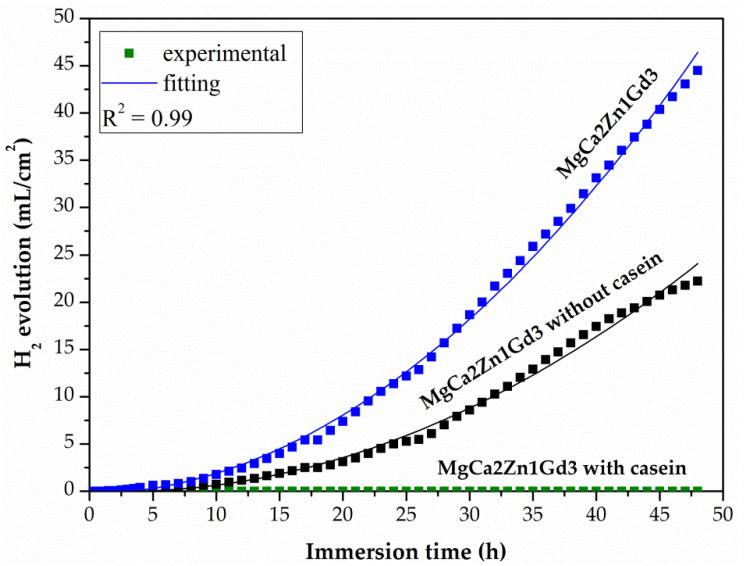
Volume of hydrogen evolution as a function of immersion time in Ringer solution at 37 °C during 48 h for the coatings applied to the MgCa2Zn1Gd3 alloy and uncoated alloy.

After potentiodynamic tests, the CN and NZ coatings applied on two Mg-based alloys and the uncoated alloys were immersed in Ringer solution at 37 °C for 48 h (for casein-free coatings (NZ) and substrate alloys) and for 216 h (for the casein coatings (CN)). The results obtained from such tests showed the real long-term corrosion behavior of biomaterials for orthopedic applications. After 48 h of immersion, the casein-free coating deposited on the MgCa2Zn1 alloy was characterized by a lower evolution value of H_2_ evolution, equal to 43.21 mL/cm^2^ compared to the uncoated alloy (the H_2_ volume for the MgCa2Zn1 was equal to 61.94 mL/cm^2^) (Figure 11). A similar situation was observed for the NZ coating applied on the MgCa2Zn1Gd3 alloy. The H_2_ volume for the casein-free coating and the uncoated alloy was 22.25 and 44.49 mL/cm^2^, respectively. Furthermore, the volume of H_2_ evolution for the NZ coating and uncoated MgCa2Zn1Gd3 alloy was significantly lower compared to the values obtained for the NZ coating deposited on the MgCa2Zn1 alloy (Figure 12). The volume of hydrogen evolved after 48 h of immersion in Ringer solution for the casein coatings applied on the two Mg-based alloys was 0 mL/cm^2^ (Figure 11 and Figure 12). The hydrogen evolution rates after immersion tests were also calculated. After 48 h of immersion in Ringer solution, the corrosion rates (v_corr_) for casein-free coatings applied to the MgCa2Zn1 and MgCa2Zn1Gd3 alloys were 0.9 and 0.46 mL/cm^2^/h, respectively. The v_corr_ for substrate alloys was slightly higher (v_corr_ of MgCa2Zn1 alloy was equal to 1.29 mL/cm^2^/h, and for the second one was 0.93 mL/cm^2^/h). 

**Figure 13:**
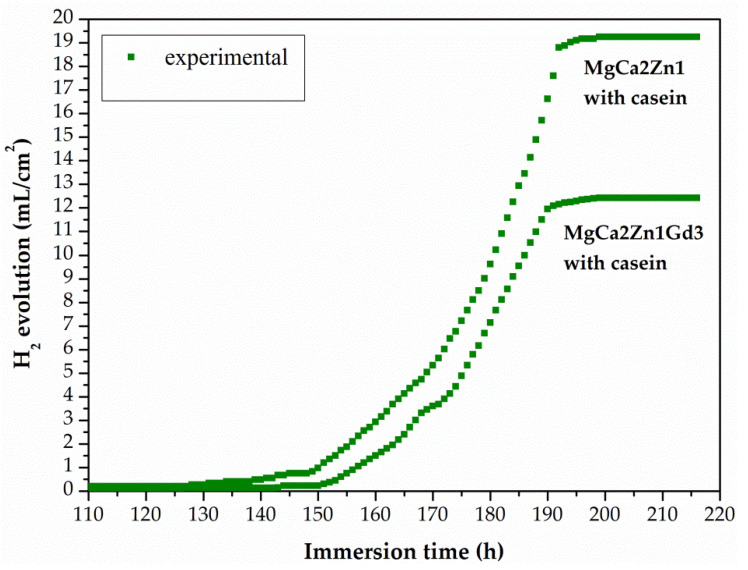
Volume of hydrogen evolution as a function of immersion time in Ringer solution at 37 °C during 216 h for the casein coatings applied to the MgCa2Zn1 and MgCa2Zn1Gd3 alloys.

The minimal increase in H_2_ volume for the casein coatings was observed until after 125 h of immersion in chloride-rich solution (Figure 13). After 190 h of immersion, the volume of H_2_ evolution for the casein coatings applied on MgCa2Zn1 and MgCa2Zn1Gd3 has stabilized and after 216 h it was 19.25 and 12.42 mL/cm^2^, respectively. In addition, the corrosion rates for these coatings were 0.09 and 0.05 mL/cm^2^/h, respectively. The results of immersion tests confirmed the electrochemical results, where the casein coating applied onto MgCa2Zn1 alloy was a cathode part of the curve and was one order lower in the current range compared to the uncoated sample (Figure 9a). This may suggest that the MgCa2Zn1 sample with casein decreases the activity of the cathode reactions (hydrogen evolution). Furthermore, the casein coatings applied to both the studied MgCa2Zn1 and MgCa2Zn1Gd3 alloys were in the same current range (Figure 9). 

In the work [41], it was found that biopolymer coatings composed of collagen and chitosan effectively retain the H_2_ gas and hence improve the corrosion resistance of the AZ31 alloy. The influence of chitosan on the improvement of corrosion resistance of the Mg-1.75Zn-0.56Ca alloy was also investigated by Dou et al. [42]. The MAO/CS composite coatings were characterized by a lower degradation rate during immersion tests.

The results of immersion tests for coatings with and without casein can be analyzed in comparison to the values obtained for the surface roughness. In the studies carried out by Nguyen et al. [39], a higher volume of evolved H_2_ was generated in samples with increasing surface roughness. 

The corrosion behavior of the casein and casein-free coatings was determined by weight loss rate after 48 h of immersion tests. The average corrosion rate, P_w_, for the casein (CN) and casein-free (NZ) coatings applied to MgCa2Zn1 was 1.1 × 10^−3^ and 2.1 × 10^−2^ mm·y^−1^, respectively. The mass loss rates for the CN and NZ coatings applied to the alloy with gadolinium addition were lower and equal to 8.6 × 10^−4^ and 9.8 × 10^−3^ mm·y^−1^, respectively. The results obtained from P_w_ correspond to a volume of hydrogen evolution for these materials. Weight loss rates also suggest that conversion coatings are resistant to corrosion in an aggressive environment.

**Figure 14:**
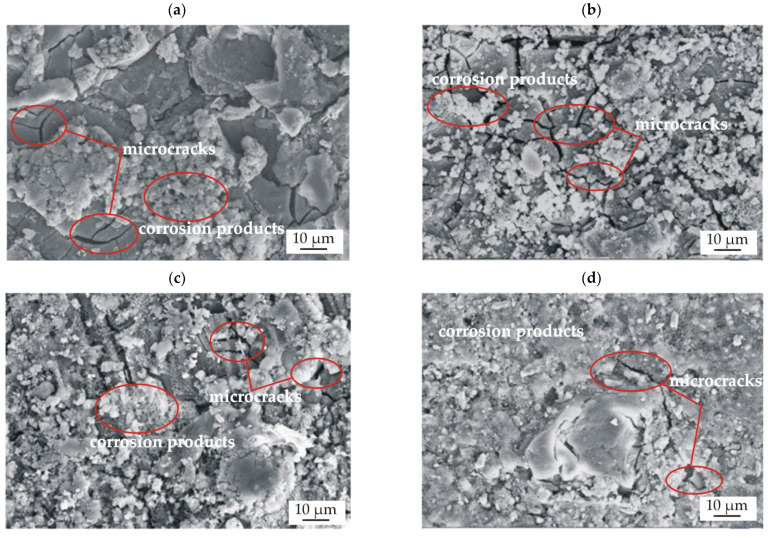
SEM images of samples’ surfaces with corrosion products for the casein-free coatings (NZ) deposited on: (**a**) MgCa2Zn1; (**b**) MgCa2Zn1Gd3, and for the casein coatings (CN) applied on: (**c**) MgCa2Zn1; (**d**) MgCa2Zn1Gd3 after 48 h of immersion in Ringer solution at 37 °C.

After immersion tests, the surface morphology of the sample was microscopically observed. The results of the SEM observations of the conversion casein coating and casein-free coating applied to the MgCa2Zn1 and MgCa2Zn1Gd3 alloys are presented in Figure 14. 

In the presented images, microcracks were observed in both studied coatings (Figure 14a–d). This phenomenon is due to dehydration during the drying of the samples. Furthermore, the surfaces of the CN and NZ coatings applied on the Mg-based alloys were covered with corrosion products (Figure 14a–d). It can be seen that the layers of corrosion products visible on the surfaces of the casein coatings were denser compared to the coatings without casein, where the corrosion products formed agglomerates. The denser layer of corrosion products can partially block the evolution of hydrogen, which was confirmed in Figure 8 and Figure 9, where the volume of evolved H_2_ for the casein coatings was 0 mL/cm^2^. 

**Figure 15:**
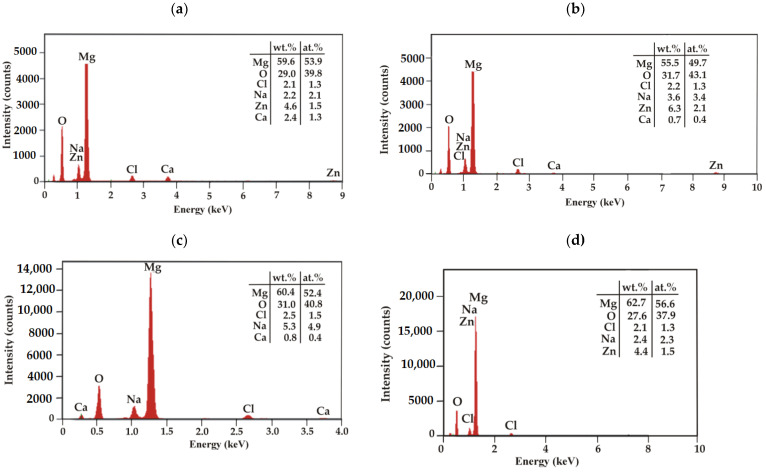
EDS analysis for corrosion products of the casein-free coatings (NZ) deposited on: (**a**) MgCa2Zn1; (**b**) MgCa2Zn1Gd3, and for the casein coatings (CN) applied on: (**c**) MgCa2Zn1; (**d**) MgCa2Zn1Gd3 after 48 h of immersion in Ringer solution at 37 °C.

EDS analysis was performed for the corrosion products of the casein and casein-free samples after 48 h of immersion in a chloride-rich solution (Figure 15). The results of the analysis indicated that these corrosion products consist mainly of Mg and O. There are also low-intensity peaks from Na, Zn, Ca, and Cl. The lower intensity of Cl^−^ on the surface of the studied samples may suggest that casein and casein-free coatings protect the substrate alloys against corrosion. More dense, less defective, and uniform coatings will prohibit electrolyte penetration through the coating, thus increasing corrosion resistance. In addition, a higher share of Mg that form hydroxides Mg(OH)_2_ in corrosion products in case of Gd-dopped Mg alloy will also help to prevent further surface dissolution.

**Figure 16:**
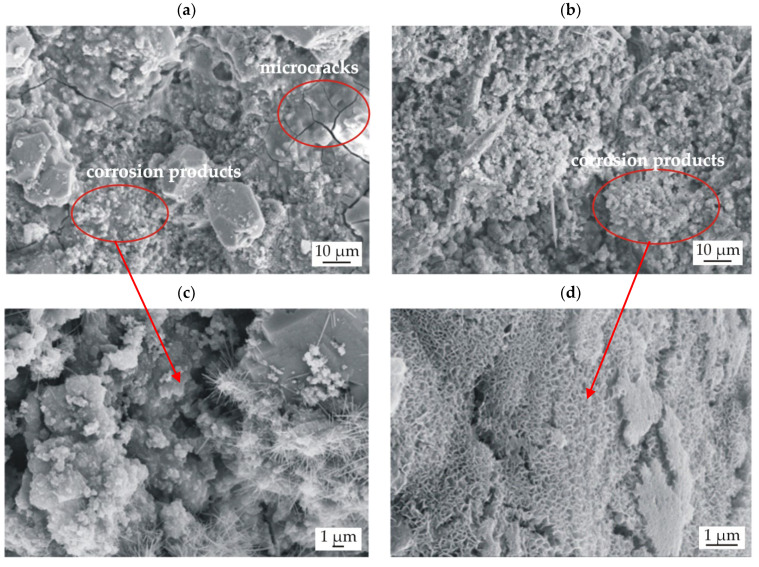
Sample surface with corrosion products for the casein coatings CN deposited on: (**a**,**c**) MgCa2Zn1 alloy; (**b**,**d**) MgCa2Zn1Gd3 alloy after 216 h of immersion in Ringer solution at 37 °C.

The surface morphology of casein coatings (CN) after 216 h of immersion in Ringer solution at 37 °C, was evolved (Figure 16). The coating surface on the MgCa2Zn1 alloy was porous and had microcracks. Some corrosion products were also observed to fall off from the surface (Figure 16a). The casein coating surface deposited on the Gd-containing Mg alloy had a denser layer of corrosion product (Figure 16b) and was less porous. This confirmed an improvement in the corrosion resistance of coated MgCa2Zn1Gd3 alloy, that is, a lower amount of hydrogen evolution, visible in Figure 13 (after 216 h of immersion, the volume of evolved H_2_ for the casein coating applied to MgCa2Zn1Gd3 was 12.42 mL/cm^2^, and for the coating applied to the second alloy, was 19.25 mL/cm^2^). Furthermore, the surface of the casein coating applied to the MgCa2Zn1 alloy was characterized by some needle-like corrosion products compared to the second surface, where lamellar-shaped corrosion products were visible (Figure 16c,d). 

## 4. Conclusions

The purpose of the work was to produce conversion coatings enriched in casein (CN) and casein-fee (NZ) on the surface of MgCa2Zn1 and MgCa2Zn1Gd3 alloys and to determine their structure and corrosion resistance in the environment of human body fluids. Based on the presented research, the following conclusions were drawn:

1. The microstructural analysis of the conversion coatings revealed that the surface morphology of the casein coatings and casein-free coatings was heterogeneous. However, the CN coatings were more compact and less porous. On the basis of the XRD analysis, it can be stated that the coatings with CN and without casein (NZ) had a crystalline structure.
2. The corrosion behavior of the casein coatings and casein-free coatings was determined by the electrochemical and immersion tests in Ringer solution at 37 °C. The results of the Tafel’s analysis showed that both coatings improved the corrosion resistance of the MgCa2Zn1 and MgCa2Zn1Gd3 alloys. It can also be observed that the casein coatings showed lower values of corrosion current density, higher corrosion potential, and polarization resistance compared with those of the casein-free coatings. The improvement in corrosion resistance was also confirmed by a lower volume of evolved H_2_ measured during immersion tests, as well as a lower mass loss for casein coatings.
3. The synergistic effect of the addition of gadolinium in the MgCaZn alloy and the formation of dense, tight conversion coatings with the addition of casein on the surface was revealed. This indicates that such a combination of substrate material and casein coating is promising for possible application as implants in orthopedics.

## Figures and Tables

**Table 1:** Surface roughness parameters of the conversion coatings on MgCa2Zn1 and MgCa2Zn1Gd3 alloys.

Substrate	Coating	Roughness Average, R_a_, µm,	Root Mean Square, R_S_, µm
MgCa2Zn1	CN	4.25	4.78
	NZ	4.35	5.01
MgCa2Zn1Gd3	CN	0.9	1.1
	NZ	1.5	1.87

**Table 2:** Electrochemical parameters of the conversion coating with casein (CN) and casein-free coating (NZ) in the MgCa2Zn1 and MgCa2Zn1Gd3 alloys.

Substrate	Coating	Corrosion Potential, E_corr_, V	Polarization Resistance, R_p_, Ω·cm^2^	Corrosion Current Density, i_corr_, μA·cm^−2^
MgCa2Zn1	-	−1.55 ± 0.03	200 ± 3	98.6 ± 4
	NZ	−1.49 ± 0.03	296 ± 4	2.7 ± 2
	CN	−1.42 ± 0.03	379 ± 3	1.23 ± 2
MgCa2Zn1Gd3	-	−1.56 ± 0.03	363.1 ± 3	56.8 ± 5
	NZ	−1.49 ± 0.03	1138 ± 4	4.02 ± 2
	CN	−1.45 ± 0.03	2137 ± 5	1.95 ± 1

**Table 3:** Impedance parameters (EIS) of the conversion coating with casein (CN) and casein-free coating (NZ) in the MgCa2Zn1 and MgCa2Zn1Gd3 alloys.

Substrate	Coating	R1, Ω·cm^2^	CPE, μF·s^n−1^·cm^−2^		R2, Ω·cm^2^	L1, H·cm^2^	R3, Ω·cm^2^	Rp(EIS), Ω·cm^2^
			Y	n				
MgCa2Zn1	NZ	21.42	24.21	0.866	620	54	70	63
	CN	21.09	16.28	0.886	1105	72	203	171
MgCa2Zn1Gd3	NZ	10.75	20.76	0.89	510	36	100	84
	CN	13.20	21.17	0.7581	2230	176	199	183

## Data Availability

The data presented in this study are available in the article.

## References

[1] Ammar H.R., Sivasankaran S., Alaboodi A.S. (2021). Investigation of the Microstructure and Compressibility of Biodegradable Fe-Mn-Cu/W/Co Nanostructured Alloy Powders Synthesized by Mechanical Alloying. Materials.

[2] Ammar H.R., Sivasankaran S., Alaboodi A.S. (2021). The influence of ball milling processing variables on the microstructure and compaction behavior of Fe–Mn–Cu alloys. Mater. Sci.-Pol..

[3] Witte F., Hort N., Vogt C., Cohen S., Kainer K.U., Willumeit R., Feyerabend F. (2008). Degradable biomaterials based on magnesium corrosion. Curr. Opin. Solid State Mater. Sci..

[4] Li N., Zheng Y. (2013). Novel Magnesium Alloys Developed for Biomedical Application: A Review. J. Mater. Sci. Technol..

[5] Zidane N., Ait Albrimi Y., Ait Addi A., Ait Akbour R., Douch J., Nahléb A., Hamdania M. (2015). Effect of Gadolinium Content on the Corrosion Behavior of Magnesium Alloys in 1 wt.% NaCl Solution. Port. Electrochim. Acta..

[6] Hort N., Huang Y., Fechner D., Störmer M., Blawert C., Witte F., Vogt C., Drücker H., Willumeit R., Kainer K.U. (2010). Magnesium alloys as implant materials—Principles of property design for Mg-RE alloys. Acta Biomater..

[7] Kania A., Nowosielski R., Gawlas-Mucha A., Babilas R. (2018). Mechanical and corrosion properties of Mg-based alloys with Gd addition. Materials.

[8] Rau J.V., Antoniac I., Filipescu M., Cotrut C., Dinescu M. (2018). Hydroxyapatite coatings on Mg-Ca alloy prepared by Pulsed Laser Deposition: Properties and corrosion resistance in Simulated Body Fluid. Ceram. Int..

[9] Jiang S., Cai S., Lin Y., Bao X., Xu G. (2019). Effect of alkali/acid pretreatment on the topography and corrosion resistance of as-deposited CaP coating on magnesium alloys. J. Alloys Compd..

[10] Acheson J.G., McKillop S., Lemoine P., Boyd A.R., Meenan B.J. (2019). Control of magnesium alloy corrosion by bioactive calcium phosphate coating: Implications for resorbable orthopaedic implants. Materialia.

[11] Shi P., Niu B., Shanshan E., Chen Y., Li Q. (2015). Preparation and characterization of PLA coating and PLA/MAO composite coatings on AZ31 magnesium alloy for improvement of corrosion resistance. Surf. Coat. Technol..

[12] Manna S., Donnell A.M., Kaval N., Marwan F. (2018). Improved design and characterization of PLGA/PLA-coated Chitosan based micro-implants for controlled release of hydrophilic drugs. Int. J. Pharm..

[13] Li L.Y., Cui L.Y., Zeng R.C., Li S.Q., Chen X.B., Zheng Y., Kannan M.B. (2018). Advances in functionalized polymer coatings on biodegradable magnesium alloys—A review. Acta Biomater..

[14] Gao Y., Jie M., Liu Y. (2017). Mechanical properties of Al_2_O_3_ ceramic coatings prepared by plasma spraying on magnesium alloy. Surf. Coat. Technol..

[15] Xu Z., Eduok U., Szpunar J. (2019). Effect of annealing temperature on the corrosion resistance of MgO coatings on Mg alloy. Surf. Coat. Technol..

[16] Liu P., Pan X., Yang W., Cai K., Chen Y. (2012). Al_2_O_3_-ZrO_2_ ceramic coatings fabricated on WE43 magnesium alloy by cathodic plasma electrolytic deposition. Mater. Lett..

[17] Mashtalyara D.V., Sinebryukhova S.L., Imshinetskiya I., Gnedenkova A., Nadaraiaa K. (2020). Hard wearproof PEO-coatings formed on Mg alloy using TiN nanoparticles. Appl. Surf. Sci..

[18] Wang S., Si N., Xia Y., Liu L. (2015). Influence of nano-SiC on microstructure and property of MAO coating formed on AZ91D magnesium alloy. Trans. Nonferrous Met. Soc. China.

[19] Priya P., Mohan Raj R., Vasanthakumara V., Raj V. (2020). Curcumin-loaded layer-by-layer folic acid and casein coated carboxymethyl cellulose/casein nanogels for treatment of skin cancer. Arab. J. Chem..

[20] Gawęcki J. (2016). Proteins in Food and Nutrition.

[21] Sikorski Z.E. (2012). Food Chemistry.

[22] Construction and Properties of Selected Polymers-Casein-Laboratory Workshop for Students. https://docplayer.pl/16394715-Budowa-i-wlasciwosci-wybranych-biopolimerow-kazeina-warsztaty-laboratoryjne-dla-uczniow-iii-lo-im-unii-lubelskiej-w-lublinie.htm.

[23] Qin L., Dong H., Mu Z., Zhang Y., Dong G. (2015). Preparation and bioactive properties of chitosan and casein phosphopeptides composite coatings for orthopedic implants. Carbohydr. Polym..

[24] Kumar B.S., Muthukumar T., Deepachitra R., Charumathy R.A., Hemalatha T., Sastry T.P. (2015). In-vitro evaluation of biphasic calcium phosphate/casein incorporated with Myristica fragrans for bone tissue engineering. Ceram. Int..

[25] Li G.Y., Lian J.S., Niu L.Y., Jiang Z.H., Jiang Q. (2006). Growth of zinc phosphate coatings on AZ91D magnesium alloy. Surf. Coat. Technol..

[26] Yabuki A., Sakai M. (2011). Self-healing coatings of inorganic particles using a pH-sensitive organic agent. Corros. Sci..

[27] Sherry A.D., Caravan P., Lenkinski R.E. (2009). A primer on gadolinium chemistry. J. Magn. Reason. Imaging.

[28] Cabral A.D., Radu T.B., de Araujo E.D., Gunning P.T. (2021). Optical chemosensors for the detection of proximally phosphorylated peptides and proteins. RSC Chem. Biol..

[29] Gomes Soares Fontes A.F. (2015). DOTA-Based Ga(III) and Gd(III) Chelates for Medical Imaging (PET, SPECT and MRI). Ph.D. Thesis.

[30] Atrens A., Shi Z., Mehreen S.U., Johnston S., Song G.L., Chen X., Pan F. (2020). Review of Mg alloy corrosion rates. J. Magnes. Alloy..

[31] Guo Y., Su Y., Gu R., Zhang Z., Li G., Lian J., Ren L. (2020). Enhanced corrosion resistance and biocompatibility of biodegradable magnesium alloy modified by calcium phos-phate/collagen coating. Surf. Coat. Technol..

[32] Cordoba L.C., Marques A., Taryba M., Caradin T., Montemor F. (2018). Hybrid coatings with collagen and chitosan for improved bioactivity of Mg alloys. Surf. Coat. Technol..

[33] Nathanael A.J., Oh T.H. (2020). Biopolymer coatings for biomedical applications. Polymers.

[34] Zhen Z., Xi T.F., Zheng Y.F. (2015). Surface modification by natural biopolymer coatings on magnesium alloys for biomedical applications. Surface Modification of Magnesium and Its Alloys for Biomedical Applications.

[35] Tiyyagura H.R., Mohan T., Pal S., Mohan M.K. (2018). Surface modification of Magnesium and its alloy as orthopedic biomaterials with biopolymers. Fundamental Biomaterials: Metals.

[36] Chen M., Chen Y., Zhang W., Zhao S., Wang J., Mao J., Wei L., Zhao Y., Huang N., Wan G. (2016). Controlling the corrosion rate and behavior of biodegradable magnesium by a surface-immobilized ultrathin 1-hydroxyethylidene-1,1-diphosphonic acid (HEDP) film. RSC Adv..

[37] Feliu S. (2020). Electrochemical Impedance Spectroscopy for the Measurement of the Corrosion Rate of Magnesium Alloys: Brief Review and Challenges. Metals.

[38] Veleva L., Fernández-Olaya M.G., Feliu S. (2018). Initial Stages of AZ31B Magnesium Alloy Degradation in Ringer’s Solution: Interpretation of EIS, Mass Loss, Hydrogen Evolution Data and Scanning Electron Microscopy Observations. Metals.

[39] Nguyen T.L., Blanquet A., Staiger M.P., Dias G.J., Woodfield T.B. (2012). On the role of surface roughness in the corrosion of pure magnesium in vitro. J. Biomed. Mater. Res. Part B.

[40] Walter K., Mathan B.K. (2011). Influence of surface roughness on the corrosion behaviour of magnesium alloy. Mater. Des..

[41] Park C.H., Pant H.R., Kim C.S. (2013). Effect on corrosion behavior of collagen film/fiber coated AZ31 magnesium alloy. Dig. J. Nanomater. Biostruct..

[42] Dou J., Wang J., Lu Y., Chen C., Yu H., Ma R.L. (2021). Bioactive MAO/CS composite coatings on Mg-Zn-Ca alloy for orthopedic applications. Prog. Org. Coat..

